# Immunopathogenesis in *Trypanosoma cruzi* infection: a role for suppressed macrophages and apoptotic cells

**DOI:** 10.3389/fimmu.2023.1244071

**Published:** 2023-08-17

**Authors:** Natália S. Vellozo, Thayane C. Matos-Silva, Marcela F. Lopes

**Affiliations:** Instituto de Biofísica Carlos Chagas Filho, Universidade Federal do Rio de Janeiro, Rio de Janeiro, RJ, Brazil

**Keywords:** apoptosis, Chagas disease, efferocytosis, fibrosis, heart pathology, inflammation, M1 macrophages, T lymphocytes

## Abstract

During *Trypanosoma cruzi* infection, macrophages phagocytose parasites and remove apoptotic cells through efferocytosis. While macrophage 1 (M1) produces proinflammatory cytokines and NO and fights infection, M2 macrophages are permissive host cells that express arginase 1 and play a role in tissue repair. The regulation of M1 and M2 phenotypes might either induce or impair macrophage-mediated immunity towards parasite control or persistence in chronic Chagas disease. Here, we highlight a key role of macrophage activation in early immune responses to *T. cruzi* that prevent escalating parasitemia, heart parasitism, and mortality during acute infection. We will discuss the mechanisms of macrophage activation and deactivation, such as T cell cytokines and efferocytosis, and how to improve macrophage-mediated immunity to prevent parasite persistence, inflammation, and the development of chagasic cardiomyopathy. Potential vaccines or therapy must enhance early T cell-macrophage crosstalk and parasite control to restrain the pathogenic outcomes of parasite-induced inflammation in the heart.

## 1 Introduction

The protozoan *Trypanosoma cruzi* infects humans and animals, establishes chronic infection, and causes Chagas disease by affecting the heart in 30% of patients (1, 2). Although 13% of the Latin American population is at risk of infection (1, 3), there is no available vaccine or effective treatment for chronic infection and established pathology (2, 4, 5). Moreover, difficulties in treating and following human patients for decades before the onset of disease symptoms, as well as the costs of human trials for neglected tropical diseases, hamper drug development, despite advances in preclinical research (3–5). Likewise, translation from drug and vaccine research towards human benefits has been delayed owing to unsolved scientific controversies about the mechanisms of Chagas disease pathogenesis (6).

Complex interactions between the parasite, the host, and the immune system underlie the development of heart pathology in Chagas disease, characterized by inflammation and fibrosis, which lead to heart malfunctioning, heart failure, and death (1, 6). Parasite infection contributes to pathology by destroying infected cells, including myocytes, and by stimulating pathogenic immune responses that kill infected cells and cause inflammation (6–8). The immune system is necessary to control *T. cruzi* infection, thereby reducing parasite spread and parasite-induced inflammation (9, 10). Nonetheless, immune responses are involved in the pathogenesis of Chagas disease by causing tissue damage and inflammation (immunopathology) (7, 8, 10, 11), whereas immunoregulatory mechanisms control immunity and/or immunopathology. The dissection of the immune response components in *T. cruz*i infection and their roles in immunopathogenesis is crucial for the development of new vaccines or therapeutic tools without stimulating immunopathology.

Macrophages play multiple and key roles as dedicated phagocytes that clear tissues from parasites and apoptotic cells, act as M1 effectors or M2 permissive host cells, and promote inflammation, tissue repair (12), and fibrosis (13). Here, we focused on the molecular mechanisms of macrophage activation and deactivation, the dual role of M1 and M2 macrophages in antiparasitic immunity, and their modulation by T cell cytokines and apoptotic cells. We consider classically activated macrophages to be M1, which express IL-12 and induced NO synthase (iNOS), produce NO, and exhibit microbicidal activity (14). In contrast, alternatively activated M2 macrophages are susceptible to parasite infection (15), express arginase 1 (Arg1) (16), and play a role in tissue repair (12). Macrophage phenotypes are complex, plastic, and interchangeable in response to diverse environmental conditions. Previously published articles provided deeper information on the full spectrum of macrophage phenotypes beyond the M1 and M2 extremes obtained under defined Th1 and Th2 cytokine conditions (17–20).

## 2 Defective M1 macrophage-mediated immunity plays a pathogenic role in Chagas disease

During acute infection, both innate and adaptive immunity are required to fight *T. cruzi* parasites in the blood, heart, and other organs (9, 10), yet parasites resist in tissue reservoirs and establish chronic infection (21). Monocytes are mobilized and recruited to the heart, where macrophages dominate early protective inflammatory responses (22). Parasite infection targets myocytes, fibroblasts, and various cell types, while macrophages continuously collect parasites released by infected cells. Macrophages can be detected in the proximity or even inside myocyte parasite nests, whereas some macrophages interact with lymphocytes or contain intracellular parasites (22, 23). In rat and mouse experimental models, macrophage depletion upon silica treatment increased parasitemia, heart parasitism, tissue damage, and mortality (23, 24), highlighting macrophage protective immunity in *T. cruzi* infection.

CD4 and CD8 T cells play a protective role by inducing NO-producing M1 macrophages (6, 9, 10). In contrast, Th2 cytokines and Arg1-expressing M2 macrophages increase susceptibility to *T. cruzi* infection (16). Therefore, the activation of M1 and M2 macrophage phenotypes might critically affect disease outcomes. Next, we will discuss how the mechanisms that govern macrophage recruitment and M1/M2 phenotypes induce either protective immunity or parasite persistence and disease progression.

Macrophage activation towards microbicidal M1 responses relies on macrophage receptors for pathogen-associated molecular patterns (PAMPs) and T cell-derived cytokines, such as IFN-γ and TNF-α, which induce iNOS expression and help to control intracellular infection. In addition, M1 macrophages secrete IL-12 and induce IFN-γ production by both NK and T cells, further enhancing type 1 responses (9, 10). Early seminal studies showed that mice deficient in IFN-γ have increased parasitemia, heart parasitism and mortality (25), even after infection with less virulent *T. cruzi* strains (26). Importantly, mice with macrophages insensitive to IFN-γ (MIIG) fail to control parasite infection *in vitro* and show increased parasitemia and mortality (27). Higher mortality, parasitemia, and nervous system inflammation were also observed upon genetic ablation of IL-12 (25). Moreover, IL-12-defective macrophages were more susceptible to *T. cruzi* infection, expressing a reduced NO response to IFN-γ and increased TGF-β production, similar to M2 macrophages (15).

A series of studies addressed the factors that influence inflammatory responses in the heart during acute *T. cruzi* infection. Silva et al. showed that mice deficient in the inflammasome components ASC/Caspase-1 and IL-1R have defective recruitment of CD11b^+^/F4/80^+^ macrophages to the heart associated with increased heart parasitism and mortality (28). In addition, a direct role of IL-1β in the induction of NO and parasite killing was suggested as part of macrophage protective responses (28). Possibly owing to unrestricted parasite infection, ASC, Caspase-1 or IL-1R knockout (KO) mice developed increased inflammation and tissue damage during late acute infection (28).

The association between the presence of the chemokine receptors CCR2/CCR5 and the chemokines CCL2/CCL3/CCL5/CXCL9 and macrophages in the hearts of *T. cruzi*-infected mice indicates that chemokines and their receptors play a role in macrophage recruitment or activation to fight infection (29–33). In agreement with this idea, mice deficient in CCR2, CCR5, CXCL9, CCL3, and CCL2 developed increased parasitemia and/or heart parasitism (29–33), whereas higher mortality was also observed in infected CCR5 and CCL2 KO mice (30, 32). Moreover, the transfer of CCR5^+^ splenocytes to CCR5 KO mice rescued macrophage recruitment to the heart and early protective inflammatory responses (30). In contrast, the transfer of CCR5^-/-^ splenocytes failed to generate macrophages in the heart. By addressing the role of CCL2 in experimental Chagas disease, Paiva et al. showed that CCL2 is expressed on the heart inflammatory foci (32). Accordingly, CCL2 KO mice have reduced inflammatory foci and macrophage activation in the heart, despite increased systemic cytokine responses secondary to uncontrolled parasitemia and tissue parasitism (32).

By studying the relevant mechanisms for parasite killing in *T. cruzi* infection, Sharma et al. showed that macrophages defective in phospholipase 2 β (PLA_2_β) have reduced NO production and increased parasite replication, whereas parasite nests are abundant in the hearts of PLA_2_β KO mice (34). Recently, Silva et al. addressed the role of phosphatidylinositol 3-kinase-γ (PI3Kγ), which is important for macrophage-mediated immunity, as highlighted by reduced NO production and increased parasite infection in PI3Kγ-defective or inhibitor-treated macrophages (35). Infected PI3Kγ KO mice exhibited increased weight loss, parasitism, heart inflammation and malfunction, tissue damage, and mortality (35). Defective downstream PI3Kγ signalling in macrophage conditional AKT1 KO mice also increased parasitism and mortality (35). Moreover, macrophages are the major players in protective immune responses mediated by PI3Kγ (35). Interestingly, infected PI3Kγ KO mice benefited from treatment with anti-inflammatory or antiparasitic drugs (35). These results suggest that both parasites and inflammatory responses contribute to disease secondary to PI3Kγ deficiency.

Next, we will discuss the downregulation/inhibition of macrophage activation in experimental BALB/c models of *T. cruzi* infection. By using CD73 KO mice and/or pharmacological CD73 inhibition, Ponce et al. showed that the CD73 ectonucleotidase deactivates macrophages during infection (36). CD73 genetic deficiency or inhibitor restored M1 responses in the heart and reduced heart parasitism, inflammation, tissue damage, and arrythmia (36). Calderon et al. addressed the role of SLAMF1, a factor that downregulates NADPH oxidase in *T. cruzi* infection (37). They show that macrophages from SLAMF1 KO mice show better control of parasite replication and that SLAMF1 KO mice have reduced arginase expression in their hearts and reduced parasitism, tissue damage, and mortality (37).

Altogether, these studies suggest a major protective role of M1-mediated immunity to *T. cruzi* during acute infection that reduces infection, mortality, and pathology (**Table 1**). Conversely, macrophage failure to fight parasites might be implicated in parasite persistence throughout chronic infection and more severe infection outcomes (38), with continuous or intermittent release of infected cell contents and antigens further insufflating inflammation and heart pathology (39).

**Table 1:** Macrophage 1 provides immunity whereas Macrophage 2 promotes infection and pathology.

Cell/molecular mechanism	Experimental infection	Macrophage findings	Infection and pathology outcomes	Ref. n°
Macrophage depletion	rats mice	monocytosis, infection	high parasitemia, tissue parasitism, tissue damage	(23, 24)
IFNR (activation)	B6 MIIG mice	MIIG M2-like macrophages	high parasitemia, tissue parasitism, inflammation, and mortality	(27)
Inflammasome Asc Casp1 IL-1R (activation)	B6 (WT)/ASC KO/Casp1 KO/IL1R KO	heart F4/80^+^ CD11b^+^ cell	high tissue parasitism and mortality, reduced early inflammation, increased pathology	(28)
CCR5 (recruitment)	B6 (WT) CCR5 KO	heart F4/80^+^ cell; cell transfer	high parasitemia, tissue parasitism, and mortality, reduced inflammation	(30)
CXCL9 (recruitment)	B6 anti-CXCL9	heart F4/80^+^ CXCL9^+^ cell	high parasitemia, tissue parasitism	(31)
CCL2 (recruitment)	B6 (WT) CCL2 KO	heart CD11b^+^ activated cell	high parasitemia, tissue parasitism, mortality, reduced inflammatory foci	(32)
CCL3 (inflammation in chronic infection)	B6 (WT) CCL3 KO Met-RANTES	CCL3^+^ splenic macrophages	high parasitemia, tissue parasitism (acute infection); reduced chronic pathology	(33)
PLA_2_β (activation)	B6 (WT) PLA_2_β KO	PLA_2_β ^-/-^ M2-like macrophages	high tissue parasitism	(34)
PI3Kγ AKT1 (activation)	B6 (WT) PI3Kγ KO AKT1-LysKO	PI3Kγ^-/-^ M2-like macrophages	high tissue parasitism and mortality, increased inflammation, tissue damage	(35)
Axl efferocytosis (inhibition)	B6 (WT) Ax KO/Mer KO	Axl^-/-^ M1-like heart iNOS^+^ cell	reduced parasitemia, heart inflammation, and fibrosis	(14)
CD73 ecto-nucleotidase (inhibition)	BALB/c CD73 KO	CD73^-/-^ M1-like heart (F4/80^+^ CD11b^+^) cell	reduced tissue parasitism and tissue damage, improved heart function	(36)
SLAMF1 (inhibition)	BALB/c SLAMF1 KO	reduced Slamf1^-/-^ M2-like	reduced tissue parasitism, mortality, and tissue damage	(37)

Importantly, the use of experimental models to follow disease development shows that the role played by protective versus pathogenic immune responses is timing dependent in acute versus chronic infection. During acute infection, CCL3-chemokine KO mice express increased parasitemia and heart parasitism, indicating that early CCL3-mediated recruitment of immune cells to the heart protects against parasite infection (33). In contrast, chronically infected mice deficient in CCL3 or treated with a chemokine receptor antagonist show reduced cardiac inflammation and tissue damage and restored heart function (33).Therefore, whereas early CCL3 expression in macrophages correlates with protective immune responses, continuous CCL3-mediated inflammation throughout chronic infection is deleterious to the host in Chagas disease (33). These results are consistent with clinical studies of chronic Chagas disease that show that heart expression of the proinflammatory cytokines IFN-γ and TNF-α, as well as chemokines, correlates with severe chagasic cardiomyopathy (1, 40).

## 3 Apoptosis underlies defective T cell help to macrophages in *T. cruzi* infection

Immunoregulatory mechanisms that defeat T cell-mediated immunity, such as the death of cytokine-producing T cells, might affect their ability to help macrophages infected with *T.* cruzi (41). By searching for defects in the immune responses underlying parasite persistence, we found that splenic T cells from infected mice proliferate less than T cells from healthy mice in response to T cell receptor (TCR) agonists (42). Moreover, during acute infection, T cells undergo activation-induced cell death, which correlates with reduced proliferative responses upon TCR engagement (43, 44). Other groups also reported increased T and B cell apoptosis in lymphoid organs during *T. cruzi* infection (45–51). Furthermore, apoptosis and defective proliferative responses occur in T cells from patients with chronic cardiac Chagas disease and heart failure (52, 53). Importantly, apoptotic cells were found in the hearts both in experimental models and in human patients (54–56).

The molecular mechanisms involved in programmed cell death have been investigated as potential targets to restore immunity during parasitic diseases (57, 58). T cells from *T. cruzi*-infected mice express increased levels of proapoptotic molecules, such as Fas (CD95) and Fas ligand (FasL, CD95L), as well as caspase-8 activity and activated caspase-3 (59–63). The extrinsic apoptotic pathway ensues during *T. cruzi* infection through FasL binding to the death receptor Fas in CD4 and CD8 T cells (59, 62). The antagonist anti-FasL mAb (62), the caspase-8 inhibitor zIETD, and the pan caspase inhibitor zVAD (60, 61) prevent activation-induced death in T cells from infected mice. T cell proliferation increases in the presence of anti-FasL and in T cells from infected FasL-deficient *gld* mutant mice (59, 62). These findings indicate that Fas-mediated apoptosis might counteract T cell expansion during infection. Moreover, activation-induced cell death and FasL-Fas expression underlie defective proliferation in patient T cells (52, 53).

Terminally differentiated effector cells undergo apoptosis to abbreviate the breadth of potentially pathogenic immune responses. Nonetheless, early apoptosis of effector T cells might curtail their ability to kill infected cells or help infected macrophages. During acute infection, antigen-specific effector CD8 T cells from infected mice express Fas and a proapoptotic phenotype (63). Likewise, CD4 T cells undergo Fas-mediated apoptosis and express a reduced ability to help infected macrophages (41, 59). The use of anti-FasL or CD4 T cells from infected *gld* mice allowed macrophages to control intracellular infection (41, 59). Pharmacological approaches used injections of anti-FasL and zVAD in *T. cruzi*-infected mice to evaluate their effects on immune responses during parasite infection (61, 62, 64). Treatment with zVAD during acute infection reduced parasitemia and apoptosis in splenocytes (61). Similarly, injection of anti-FasL reduced peak parasitemia and apoptosis in splenic CD8 T cells (62). Moreover, in both cases, infected mice had increased cytokine responses, and their macrophages expressed an improved ability to control parasite infection (61, 62, 64).

To directly address whether CD8 T cells cooperate with macrophages to fight *T. cruzi* parasites, splenic or peritoneal CD8 T cells and macrophages from infected mice were cocultured to evaluate IFN-γ production, T cell apoptosis and macrophage responses (64). Upon T cell activation, the failure of macrophages to produce NO and restrict parasite infection correlated with increased CD8 T cell apoptosis and development of the M2 phenotype (64). Treatment *in vitro* or *in vivo* with anti-FasL reduced T cell apoptosis, improved M1 responses, and restored macrophage-mediated immunity to *T. cruzi* infection (64). Altogether, these results suggest that the induction of T cell apoptosis during infection contributes to defective T cell and macrophage immune responses, allowing a permissive environment for parasite persistence towards the development of chronic infection.

Although these studies are useful as a proof of principle that apoptosis negatively regulates protective immune responses mediated by T cells, pharmacological and genetic ablation of apoptosis pathways during infection opens a “Pandora box” of undesirable effects such as the onset of autoimmunity in *gld*/*lpr* models (65–67) or increased inflammation in the hearts of *T. cruzi*-infected mice treated with anti-FasL (58). Genetic inhibition of the FasL-Fas or caspase-8 pathways also dysregulated Th2 cytokine responses and increased parasite infection (48, 59, 60). Moreover, these studies revealed that caspase-8 is also required for CD8 T cell expansion during *T. cruzi* infection (60). Finally, Bim-deleted mice are more susceptible to *T. cruzi* infection, most likely owing to defective macrophage and T cell responses (68). Therefore, the translation of apoptosis inhibition into treatment for chronic diseases is unlikely so far. Nonetheless, vaccine approaches might be useful to prevent the development of proapoptotic T cells, thereby improving antiparasitic immune responses (63).

## 4 Efferocytosis suppresses macrophage-mediated immunity

Apoptotic cells express ‘eat me signals’ in the outer membrane, such as phosphatidylserine, allowing their detection and clearance by phagocytes, a process named efferocytosis. The phagocytosis and dismounting of apoptotic cells prevent their accumulation in tissues and the release of proinflammatory cell content through secondary necrosis (69, 70). Several receptors might be involved in the detection of phosphatidylserine and phagocytosis (69, 70). In addition, apoptotic cells actively signal through macrophage receptors and induce anti-inflammatory responses (71, 72). How these receptors cooperate with each other and engage signaling pathways to convey proper responses to apoptotic cells is a complex scenario under investigation (72–74).

By using electron and light microscopy and immunofluorescence, we detected apoptotic lymphocytes inside macrophages from the spleen (75) and peritoneum (64) during *T. cruzi* infection. We found an apoptotic CD8 T cell inside a peritoneal macrophage and macrophages containing both parasites and apoptotic bodies during *T. cruzi* infection (64). To investigate how efferocytosis directly affects macrophage ability to fight *T. cruzi* parasites, we added apoptotic T cells to peritoneal macrophages from infected mice and evaluated endogenous infection by assessing parasites released from macrophages (76). Treatment with apoptotic but not necrotic cells exacerbated *T. cruzi* infection within macrophages and increased parasitemia upon injection in infected mice (76). Moreover, the receptor α_v_β_3_ mediates apoptotic cell uptake and macrophage responses, such as the production of TGF-β and PGE_2_ and ornithine decarboxylase activity (76). These findings indicated that efferocytosis diverts L-arginine metabolism towards polyamine synthesis, which favors parasite survival and replication (76).

To address the role of efferocytosis during *T. cruzi* infection, we employed two mouse strains individually defective in Axl and Mer, two out of three TAM receptors involved in efferocytosis (14). Double Mer^-/-^Axl^-/-^ and single Mer-defective strains have been previously used in *Leishmania* infection to show that infected neutrophils transfer *Leishmania* parasites to macrophages or DCs through efferocytosis and reduce macrophage and T cell responses (77, 78).

By employing bone marrow-derived macrophages treated with a TAM receptor inhibitor or Mer- and Axl-defective macrophages, we investigated macrophage responses to T cells from *T. cruzi*-infected mice, which bear both effector activity and proapoptotic cells (14). Efferocytosis of apoptotic T cells was blocked by a TAM receptor inhibitor, whereas Mer or Axl deficiency partially inhibited efferocytosis (14). Remarkably, TAM inhibition and Axl but not Mer deficiency improved M1 responses to T cells from *T. cruzi*-infected mice (14). These results indicate that Axl downregulates M1 macrophages, despite predominant Mer expression and the major role of Mer in efferocytosis. More importantly, Axl suppressed the expression of iNOS, NO production, and the ability of macrophages to fight parasite infection (14).

Zagorska et al. (79) previously reported that Mer and Axl play distinct roles in macrophage function. At homeostasis, constitutive Mer expression is important for the clearance of continuously generated apoptotic cells and to prevent inflammatory responses upon secondary necrosis. During immune responses, macrophage activation induces Axl expression to counteract increased inflammatory responses (79). To address the role of Axl in the removal of apoptotic cells during *T. cruzi* infection, we treated peritoneal macrophages from infected WT and Axl^-/-^ mice with fluorescent apoptotic T cells. Detection of apoptotic cells undergoing efferocytosis was reduced in Axl-defective macrophages from infected mice (14). In addition, the overaccumulation of splenic apoptotic T cells in infected Axl^-/-^ mice is further evidence of defective Axl-mediated efferocytosis (14).

**Figure 1:**
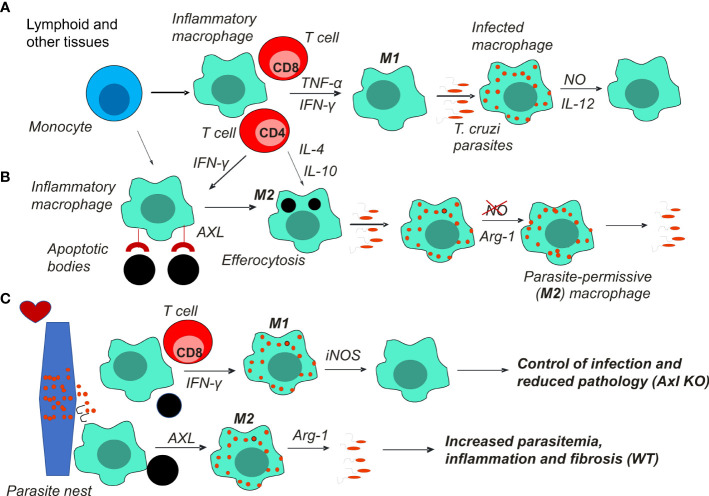
Macrophages play a key role in *T. cruzi* infection and Chagas disease pathology. **(A)** Monocytes recruited to lymphoid and other tissues differentiate into M1 macrophages under stimulation by the type 1 cytokines IFN-γ and TNF-α produced by T cells. M1 macrophages produce NO to control parasite infection and proinflammatory cytokines, such as IL-12 and TNF-α. **(B)** Alternatively, Th2 cytokines or the uptake of apoptotic cells (efferocytosis) induce parasite permissive M2 host cells that express Arg1 and fail to produce NO. The balance between M1 and M2 macrophages determines parasite control or escape and the development of chronic infection. **(C)** Pseudocysts of parasites within myocytes can rupture and release parasites in the heart. Foci of inflammatory macrophages clear heart tissues from parasites and apoptotic cells. M1 macrophages that control parasites prevent further inflammation and fibrosis, thereby reducing heart pathology. Otherwise, suppressed macrophages promote infection, inflammation, and fibrosis in the heart. Axl receptor-mediated efferocytosis might underlie macrophage suppression by apoptotic cells.

During *T. cruzi* infection, Axl^-/-^ mice expressed reduced peak parasitemia coupled with increased M1 responses in the spleen, peritoneum, and heart tissues (14). Furthermore, hearts collected from infected Axl^-/-^ but not Mer^-/-^ mice had reduced inflammation and fibrosis characteristics of heart pathology in Chagas disease (14). Overall, these findings indicate that Axl disrupts M1-mediated immunity to *T. cruzi*, fostering inflammatory responses and fibrosis in the heart (**Figure 1**).

## 5 Concluding remarks

The development of antiparasitic therapy to treat *T. cruzi* infection has progressed recently (4). Likewise, the new vaccine generations tested for COVID-19 will help vaccine development for Chagas disease and other neglected diseases. Since early macrophage responses to T cell cytokines and apoptotic cells control macrophage M1/M2 responses and disease outcomes, the use of appropriate vaccine adjuvants to target macrophage activation and dampen regulatory circuits might upregulate early protective responses. In agreement with this, experimental vaccines induced protective cytokine responses by T cells and macrophages and reduced parasitemia, tissue parasitism, heart pathology, and mortality (80, 81). Improved T cell and macrophage responses induced upon vaccination in endemic areas might help to prevent the formation of larger parasite reservoirs in tissues and intermittent cycles of infection that underly inflammatory responses (38, 39) and the pathogenesis of Chagas disease.

## Author contributions

NV cowrote the manuscript; TM-S discussed and reviewed the manuscript; ML analysed the literature and wrote the manuscript. All authors contributed to the article and approved the submitted version.
